# Cell surface GRP78: a potential mechanism of therapeutic resistant tumors

**DOI:** 10.1186/s12935-023-02931-9

**Published:** 2023-05-23

**Authors:** Rajalakshmi Amaresan, Udhayakumar Gopal

**Affiliations:** 1 Department of Zoology, Auxilium College, Gandhi Nagar, Vellore, 632 006 Tamil Nadu India; 2 Department of Neurosurgery, University of Mississippi Medical Center, Jackson, MS 39216 USA

**Keywords:** CS-GRP78, Chemoresitance, Radioresistance, Drug resistance, ER-stress, C38 monoclonal antibody, anti-GRP78 autoantibody

## Abstract

GRP78 is a protein that acts as a chaperone within the endoplasmic reticulum (ER) and has multiple functions. It is induced by stress and abets cells from survival. Despite, multiple Stress conditions like ER, chronic psychological and nutritional stress, hypoxia, chemotherapy, radiation therapy, and drug resistance induce cell surface GRP78 (CS-GRP78) expression in cancer cells. Further, CS-GRP78 is associated with increased malignancy and resistance to anti-cancer therapies and is considered a high-value druggable target. Recent preclinical research suggests that targeting CS-GRP78 with anti-GRP78 monoclonal antibodies (Mab) in combination with other agents may be effective in reversing the failure of chemotherapy, radiotherapy, or targeted therapies and increasing the efficacy of solid tumors treatment. This article will review recent evidence on the role of CS-GRP78 in developing resistance to anti-cancer treatments and the potential benefits of combining anti-GRP78 Mab with other cancer therapies for specific patient populations. Furthermore, our limited understanding of how CS-GRP78 regulated in human studies is a major drawback for designing effective CS-GRP78-targeted therapies. Hence, more research is still warranted to translate these potential therapies into clinical applications.

## Introduction

Endoplasmic reticulum stress (ERS) in solid tumours is caused by an imbalance in protein synthesis, folding, and secretion and abnormal glycosylation, which is exacerbated by microenvironmental factors such as lack of nutrients, hypoxia, excessive oxidative stress, and long-term viral infection [1, 2]. Tumour cells activate the unfolded protein response (UPR) to cope with ERS, which is controlled by three ER transmembrane sensors: inositol-requiring transmembrane kinase/endoribonuclease 1α (IRE1α), activating transcription factor 6 (ATF6), and protein kinase R–like ER kinase (PERK). These sensors are kept inactive in normal cells by their association with the ER chaperone protein glucose-regulated protein 78 (GRP78), also known as binding immunoglobulin protein/Heat shock protein family A (HSP70) member 5 (BiP/HSPA5) [3]. When there is an excessive load of client proteins, GRP78 dissociates and binds unfolded or misfolded proteins, allowing the sensors to activate signalling pathways that restore protein folding and secretion [4]. A prolonged state of ERS activates the transcription factor CHOP (CCAAT/enhancer-binding protein homologous protein), potentially leading to apoptosis [5]. Typical of ERS factors, GRP78 may also regulate the transcription of genes related to cell survival and apoptosis. This highlights the importance of comprehending GRP78’s multifaceted impact on cell proliferation and survival.

The multifunctional protein GRP78 is present in numerous forms and locations including intracellular, cell surface, secreted, and circulating. Although it primarily resides within the ER, it can also be found in other cellular compartments such as mitochondria and nuclei. Initial investigations proposed that cell surface GRP78 (CS-GRP78) is a transmembrane protein [6]. However, recent studies have shown that it can also function as a non-transmembrane peripheral membrane protein [7]. Additionally, CS-GRP78 has been shown to associate with several membrane proteins, including glycosylphosphatidylinositol (GPI)-anchored membrane proteins [7]. Although the exact mechanism by which GRP78 is translocated to the cell surface is still obscure, several mechanisms have been proposed. Under normal physiological conditions, GRP78 is retained in the ER through interactions with the KDEL sequence and the KDEL receptor. However, under conditions of ER stress, GRP78 can escape ER retention by separating from the KDEL receptor and passing through the Golgi apparatus to the cell surface [8, 9]. Several proteins, including GIV, Par-4, MTJ-1, and CD44v, interact with GRP78 in the ER, facilitating GRP78 translocation to the cell surface [10]. Alternatively, GRP78 can escape the ER with the assistance of DNAJC3 within an ER-derived vesicle and undergo endosome formation and fusion mediated by several Rab GTPases [11]. Recent studies have reported that the acetylation status of GRP78 is also essential for the ability of GRP78 to localize on the cell surface [12]. In addition, GRP78 has been found in extracellular vesicles (EVs) and released into extracellular fluids, where it interacts with recipient cells [13]. These studies suggest that the localization of CS-GRP78 may vary depending on the type of cell or tissue in which it is found. Further, it may be related to the cells’ physiological functions, like various stress stimuli and the type of proteins interacting with GRP78 in the ER and on the cell surface.

The different locations of GRP78 are linked to different signaling pathways that modulate apoptosis, cell invasion, and metastasis [14, 15]. Cancer cells are often subjected to ER stress under targeted therapies such as chemotherapy, radiotherapy, and drug therapy which act in part by inducing the ERS mediated UPR signaling pathway leading to resistance [16]. When GRP78, the master regulator of the UPR, is under cellular stress, it translocates to the cell surface and acts as a signaling receptor for various ligands, which promotes metastatic disease and therapeutic resistance, worsening patient outcomes [17]. Elevated levels of CS-GRP78 have been identified in various types of cancer, including prostate [6], pancreatic [18], breast [19], lung [20], gastric [21], glioma [22], ovarian [23], hepatocellular [24], esophageal [25], head and neck [26], fibrosarcoma [27], melanoma [22], renal [28], and endometrial [29]. In addition, elevated levels have been linked to a higher pathological grade, more aggressive characteristics, recurrent disease, and poor survival outcomes [30, 31]. Furthermore, high levels of GRP78 autoantibodies (Aab) directed against the NH2-terminal domain of GRP78 have been discovered in the serum of cancer patients. They are considered as a reliable marker for recurrent and metastatic progression and poor survival [6]. Interestingly, CS-GRP78 has been established as a novel regulator of PI3K signaling both in vitro and in vivo [32–35]. There are various ways in which CS-GRP78 may influence AKT activation. One known mechanism is that Aab targeting the N-terminus of GRP78 can mimic the effects of activated α_2_-Macroglobulin (α_2_M*) as a ligand. This triggers PI3K-dependent activation of AKT and promotes cellular proliferation in vitro [6, 36]. Conversely, the carboxyl-terminal domain antibody is an antagonist of α_2_M* and reduces AKT phosphorylation induced by α_2_M* [36]. Additionally, a recent study has shown that a specific monoclonal antibody (Mab) targeting CS-GRP78 is effective in suppressing PI3K/AKT signaling, tumor growth, and metastasis in multiple cancer models [37]. Notably, the CS-GRP78 protein activates various signaling pathways to influence a group of epigenetically altered genes in cancer. It is important to note that the transcriptional dysregulation caused by CS-GRP78 in cancer can occur from genetic changes, either indirectly through signaling factors such as NF-KB, STAT3, and SMAD influencing transcriptional control, or directly through genetic changes in genes themselves, such as c-Myc, YAP/TAZ, and histone acetylation [18, 22, 38, 39]. A recent study suggests that chronic psychological stress may contribute to the development of breast cancer stem cells (CSCs) through the activation of the ERS protein GRP78 and its interaction with LRP5 on the surface of cells. This connection between chronic stress and the growth of CSCs through CS-GRP78 may open new opportunities for therapy that address the underlying stress condition, potentially improving outcomes for breast cancer patients [40]. Therefore, GRP78 cell surface expression may serve as a biomarker for tumor behavior and treatment response.

In recent years, CS-GRP78 has emerged as an essential factor in resistance to various targeted therapies and correlates inversely with the sensitivity of cells to cytotoxic agents. Currently, there are several well-documented cases in which CS-GRP78 acts as a crucial mediator of therapeutic resistance through its role in tumor cell survival signaling, its impact on the tumor microenvironment or both. Interestingly, functional connections between CS-GRP78 and the bypassed target in physiological situations have been identified, indicating that resistance originates from pre-existing mechanisms. Recently, targeting CS-GRP78 with Mab could be an effective strategy to overcome adaptive resistance to chemotherapy [33] radiotherapy [18] or targeted therapies [41]. It is well-established that cancer therapies can induce ERS and lead to the expression of CS-GRP78, thereby promoting therapeutic resistance as a primary effect. This is likely to have a major impact on the outcomes for patients with advanced cancers. Hence, combination therapies targeting the role of CS-GRP78 in mitigating therapeutic stress to induce cancer cell death are necessary. As a result, there is increasing interest in using Mabs targeting CS-GRP78 in combination with other therapies in clinical trials. This review discusses recent findings that highlight the role of CS-GRP78 in regulating inherent and acquired resistance to anticancer therapies.

## CS-GRP78 mediates resistance to therapy

### Role of CS-GRP78 in chemoresistance

GRP78 has been explored as a therapeutic target in cancer and may be a promising objective for sensitizing to chemotherapy. Recently, there has been much interest in studying GRP78 as a critical mediator of cytoprotection and chemoresistance through its localization on the cell surface and downstream signaling. Cancer cells may become more resistant to chemotherapy and increase their survival when their ER regulation is altered under stress. As a result, targeting this response has become a potential therapeutic strategy for various cancers. The accumulation of GRP78 and its movement to the cell surface can be caused by microenvironmental, oncogenic, or therapeutic stress, leading to ERS and UPR. Gemcitabine, a chemotherapy drug facilitates the interaction of stress-response proteins, CLPTM1L with GRP78 which is then relocated to the cell surface through ERS pathway. Anti-CLPTM1L antibodies have been shown to inhibit anchorage-independent growth, GRP78-mediated chemoresistance, and AKT phosphorylation in pancreatic tumors, suggesting a unique and potentially targetable mechanism of cytoprotection and resistance to chemotherapy [42]. Cyclic AMP responsive element binding protein 3-like 1 (CREB3L1) is a part of the UPR and a breast cancer metastasis suppressor. It promotes the expression of target genes, such as GRP78, through its action on cyclic AMP. Studies have shown that chemotherapy drugs Doxorubicin (DOX) and Paclitaxel (Tx) can activate CREB3L1 and increase CS-GRP78 expression in triple-negative breast cancer cells (TNBC), reducing their ability to migrate and form metastases. Knockout of CREB3L1 using CRISPR/Cas9 technology has been shown to decrease GRP78 expression and eliminate the metastasis-suppressing function of CREB3L1. In mouse models, the absence of CREB3L1 leads to increased metastasis, which cannot be prevented by chemotherapy [43]. These studies prove that chemotherapy drugs regulate the ERS and UPR, which then activating the CS-GRP78 signaling axis.

GRP78 plays a significant role in promoting tumor-initiating cell populations (tumor stem cells) in pancreatic cancer [15]. Chemotherapy regimens DOX and Tx work partly by activating the UPR in cancer cells through the ERS pathway [44–46]. Although these drugs have demonstrated the ability to hinder the migration of differentiating cells, there may be a subset of tumor stem cells that express CS-GRP78 that are unaffected [15]. Studies have also shown that residual breast tumor cells after neoadjuvant therapy often have positive CS-GRP78 expression, suggesting a resistant clone with stemness features that is insensitive to chemotherapy [47, 15]. Therefore, treatment for metastatic TNBC should target the transition of CREB3L1 and CS-GRP78 expression through chemotherapy in addition to GRP78-targeted drugs to eliminate tumor-initiating clones. The residual tumor after neoadjuvant systemic therapy is more likely to exhibit positive CS-GRP78 expression than pre-treatment tissue, which may be due to the activation of the ERS response and the induction of GRP78 to translocate into the cell surface during chemotherapy [48]. As previously shown these cells may also exhibit reduced proliferation and metastasis [48, 49]. However, high GRP78 expression has also been found to have a predictive value for resistance to DOX in some studies, but not all, and may be specifically resistant to topoisomerase inhibitors [50, 51, 19, 52–54]. GRP78-targeted nanodroplets (NDs), known as SP94-DOX-NDs, encapsulate DOX and are modified with SP94 ultrasonic technology, serving as a nano platform with both precise targeting ability for chemotherapy and ultrasound imaging capability. The new oncotherapy nanodroplets offer a combination of imaging diagnosis and high therapeutic efficacy, providing a cutting-edge approach to precise cancer treatment. The newly developed SP94-DOX-NDs can serve as both a drug delivery system and non-invasive contrast agents, enabling site- and time-specific drug release through ultrasound irradiation for treating castration-resistant prostate cancer (CRPC) [55].

Cancer cells can enter a dormant state, known as “tumor dormancy,“ where cell division stops, and the cells enter a quiescent phase in the G0/G1 phase of the cell cycle [56]. These dormant cells can reactivate when conditions are favorable for cell growth and metabolism. This phenomenon is seen in various types of cancer, including early stages of tumor growth and distant metastases. The reactivation of dormant cells significantly contributes to cancer recurrence following chemotherapy and radiation therapy [57]. Recent evidence suggests a strong connection between the UPR and tumor dormancy. Studies have shown that recurrent tumors often have high ATF6 [58], associated with a poor prognosis for colon tumors [59]. High ATF6 expression is also commonly found in metastatic lesions rather than the primary tumor [60]. This may be because ATF6 is active in dormant cells, as seen in human squamous carcinoma models. In these tumors, silencing ATF6 reduces cell survival and tumor growth by downregulating adaptive pathways such as mTOR [61]. Additionally, ATF6 controls the expression of specific proteins associated with tumor transformation [62] and increased chemoresistance [63]. In glioblastoma, ATF6 also regulates the expression of several pro-oncogenic proteins like GRP78 and Notch1 that contribute to resistance to radiotherapy. These findings may shed light on why recurrent tumors are frequently resistant to subsequent rounds of chemotherapy. Different components of the UPR can also influence quiescence through various signaling pathways. For instance, in prostate cancer, IRE1 can regulate cyclin D1 expression in an XBP1-dependent way, influencing cell cycle progression and proliferation [64]. While current data is mainly consistent, growing evidence suggests that the UPR regulates cancer cell dormancy. Additionally, PERK negatively regulates cyclin D1 expression and induces cell cycle arrest in G1, which may be linked to tumor dormancy [65]. Furthermore, both PERK activation and eIF2α phosphorylation contribute to the drug resistance of dormant cells [66]. Hence, further studies are required to explore the role of CS-GRP78 in cancer cell dormancy, which will reveal complex metastasis biology and might turn into a novel therapeutic target.

Despite advancements in treatment options, the emergence of CRPC and resistance to chemotherapy remains a prevalent issue. One potential strategy to address this is using anti-KDEL antibodies, which can target cancer cells in CRPC by recognizing the COOH-terminal domain of GRP78 present on the surface of these cells. This can improve the effectiveness of chemotherapy. Polymeric nanoparticles (NPs) modified with an anti-KDEL molecule and loaded with Tx can specifically target prostate cancer cells that express GRP78. The sensitivity to Tx was evaluated in three different prostate cell lines: PNT1B, a normal cell line, PC3, a cancer cell line with low expression of GRP78 on its surface, and DU145, a cancer cell line with high expression of GRP78 on its surface. The targeted formulation greatly enhances the sensitivity of the cell line to Tx when it expresses GRP78 on its surface, compared to other treatments. This implies that GRP78 targeted therapy can significantly impact castrate resistant tumours that express GRP78 on their cell surface [67]. Under genotoxic stress, GRP78 is moved to the cancer cell surface and interacts with other proteins like Cripto-1 to block pro-apoptotic pathways [68]. The abnormal expression of GRP78 in the ER of cancer cells is linked to chemotherapy resistance in many types of cancer [16]. GRP78 may bind to and stimulate an anti-apoptotic receptor on the cell surface, such as Cripto-1, which can hinder pro-apoptotic signals triggered by the UPR [68]. Likewise, CS-GRP78/Cripto-1 complex induces a strong pro-survival signal by activating the ERK and perhaps the AKT signaling pathway [69]. Cisplatin is a chemotherapy drug associated with a high rate of resistance. Par-4 (prostate apoptosis response 4) is a tumor suppressor that can make tumor cells more sensitive to chemotherapy. Co-localizing Par-4 with CS-GRP78 leads to high expression of ER proteins ATF4 and BAX, activating the ER apoptosis pathway. When treated with Par-4 and cisplatin, the growth of xenografts in mice is repressed. Par-4 expression and cisplatin synergize in SK-NEP-1 cells, inhibiting cell growth and inducing apoptosis. Upregulation of Par-4 expression is essential for the movement of GRP78 to the cell surface and apoptosis of cancer cells in both in vitro and in vivo settings. The simultaneous use of ectopic Par-4 and cisplatin hindered the growth of human Wilms’ tumor cells both in vitro and in vivo. The impact may be related to the initiation of the ER apoptosis pathway and the interaction between extracellular Par-4 and GRP78 [28]. Therefore, this combined therapeutic approach may be a promising option for treating Wilms’ tumor.

GRP78 plays a vital role in the pro-survival pathway of the UPR signaling network. HSPA5 is highly expressed in B-lineage acute lymphoblastic leukemia (ALL) and its expression increases at relapse. A DOX-conjugated cell-penetrating cyclic anti-HSPA5 peptide can effectively kill chemotherapy-resistant B-lineage ALL cells. A polyphenolic compound found in green tea called epigallocatechin gallate (EGCG) targets the ATP-binding domain of HSPA5 and can overcome resistance to the standard anti-leukemic drug vincristine by inhibiting the anti-apoptotic function of HSPA5 to make B-lineage ALL cells more sensitive to chemotherapy [70–73]. Besides, EGCG also increases GRP78 in the ER, and induces ATF4, spliced XBP1, CHOP, and EDEM expressions, combined with a reduction of CS-GRP78 and a rise in caspase 3 and 8 activities. EGCG’s impact on malignant mesothelioma (MMe) cells includes increasing the amount of GRP78 in the ER, disrupting its function, and transforming normal UPR into pro-apoptotic ERS. This suggests that EGCG may have therapeutic potential as a co-drug in treating MMe, capable of overcoming resistance to conventional drugs at safe doses [74]. Isoliquiritigenin, a chalcone-type flavonoid isolated from liquorice root is reported to reduce the protein expression of mRNA and membrane GRP78, a critical mediator of tumour biology. Overexpression of GRP78 impacted oral squamous cell carcinomas (OSCC), cancer stem cell markers; it reversed the inhibitory effect of isoliquiritigenin on these cell markers. Likewise, in nude mice bearing OSCC xenografts, isoliquiritigenin retarded the tumor growth. Inclusively, It is proposed that isoliquiritigenin, a natural compound, can be an effective addition to chemotherapy for OSCC [75]. Therefore, targeting CS-GRP78 with a combination of chemotherapy and natural compounds could be an effective cancer treatment.

Cellular senescence, a state of permanent cell growth arrest, has been linked to resistance to chemotherapy. Cisplatin, a standard chemotherapy drug, can cause changes in the expression of the ATM gene at sub-toxic concentrations, leading to cellular senescence. Cells that have undergone senescence due to cisplatin treatment show increased cell surface expression of a protein complex called GRP78/MTJ1. This complex acts as a form of GRP78, which switches from a protein folding regulator to a signalling receptor. When activated, this receptor can trigger the Akt signalling pathway and influence stem-like characteristics by increasing the expression of transcription factors essential for self-renewal, including Nanog, Oct4, and Sox2, which drive senescence. This finding may aid in developing new treatment strategies [20]. Treating glioma through traditional chemotherapy is challenging in clinical settings because of the presence of two barriers, the blood-brain barrier (BBB) and the blood-brain tumour barrier (BBTB), that impede most chemotherapy drugs from reaching brain tumours. To overcome this challenge, an optimal drug delivery system is required to cross the BBB and BBTB efficiently and deliver therapeutics to glioma cells with high specificity. Dual NLCs, which are nanostructured lipid carriers modified with two stable D-peptides D8 (targeting nicotine acetylcholine receptors) and RI-VAP (binding to CS-GRP78) were found to specifically internalize into blood-brain endothelial cells, tumour neovascular endothelial cells and glioma cells with high efficiency and can effectively penetrate through *in-vitro* BBB and BBTB models. Bortezomib (BTZ) loaded Dual NLCs can effectively deliver BTZ to glioma cells, resulting in the highest therapeutic efficiency through inducing apoptosis, prolonged survival rate and efficient anti-glioma behaviour [76]. This suggests that Dual NLCs have great potential as a brain cancer treatment with promising therapeutic outcomes.

**Fig. 1:**
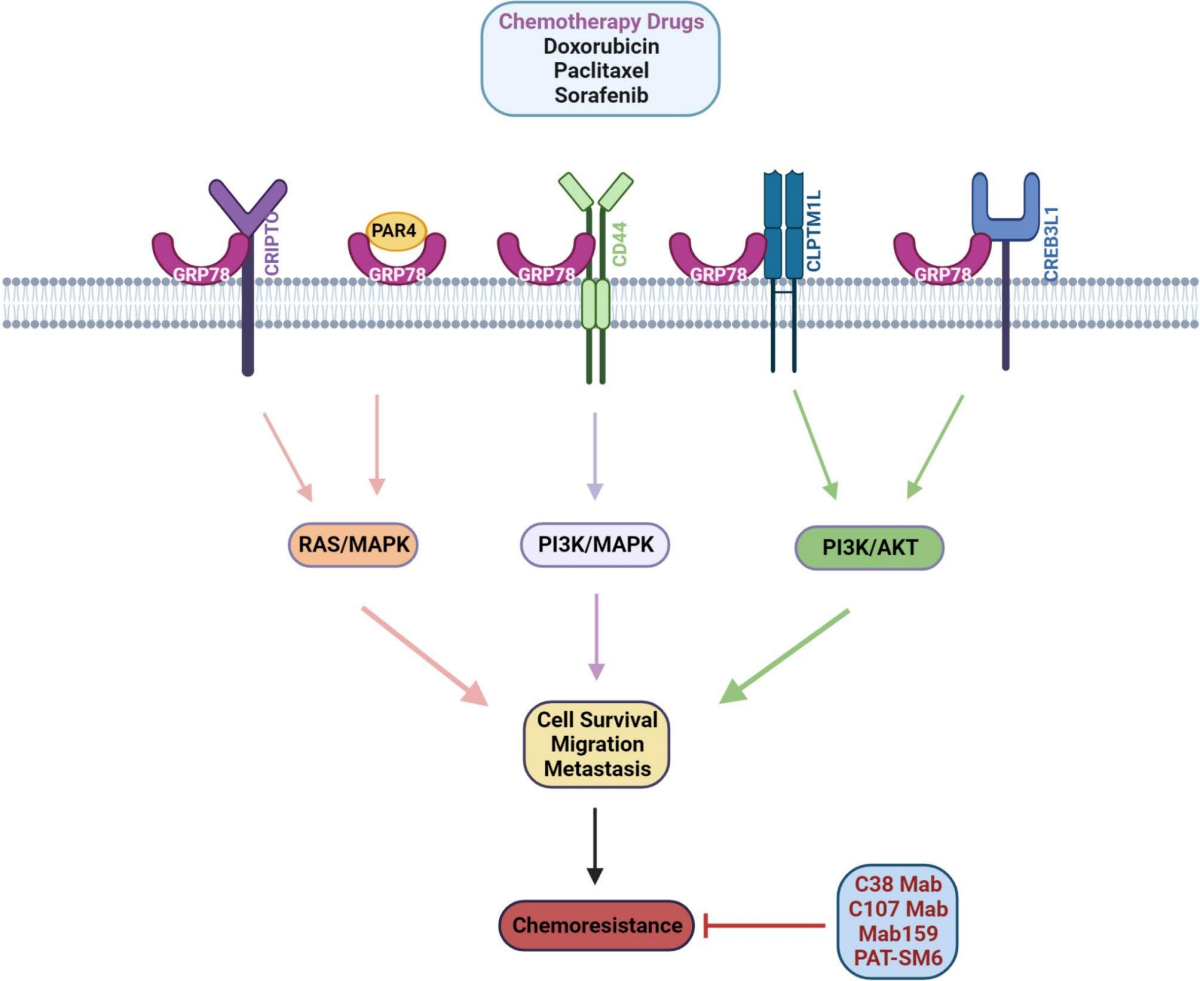
**CS-GRP78 mediates resistance to chemotherapy and creating opportunities for combination therapy.** Chemotherapy drugs induces cell surface expression of GRP78 and several other stress proteins and functions as coreceptors or ligands. Chemoresistance appears to be reinforced by CS-GRP78 through common downstream signaling pathways, such as RAS/MAPK and PI3K/AKT. Targeting CS-GRP78 with anti-GRP78 Mab is the most promising strategy to enhance anticancer activity and aid chemotherapy resistance

Distinct tumors but not myeloma cells can inhibit BTZ by secretion of GRP78 on proteasome inhibition, thus manifesting a hitherto unknown mechanism of resistance to BTZ. Indeed, many BTZ-resistant solid tumor cell lines, such as PC-3 and HRT-18, could secrete large amounts of GRP78, except for myeloma cell lines like U266 and OPM-2. Resistance to BTZ in endothelial cells and OPM-2 myeloma cells was provided by recombinant GRP78. Thus, silencing the expression of the GRP78 gene in tumor cells and immunodepleting GRP78 protein from the supernatants of tumor cells returned the sensitivity to BTZ. In these cells, GRP78 did not form a complex with BTZ or bind to it, but rather activated pro-survival signals by phosphorylating extracellular signal-related kinase and blocking the p53-mediated expression of pro-apoptotic Bok and Noxa proteins. Therefore, certain solid tumor cells can secrete GRP78 into the tumor microenvironment, revealing a previously unknown resistance mechanism to BTZ [77]. Recent studies have demonstrated that 5-fluorouracil-1-acetic acid (5-FA), a long-circulating drug carrier, can overcome the challenges of low solubility and dose-limiting side effects of rapalogues in breast cancer treatment by incorporating rapamycin-binding domains with elastin-like polypeptides (ELPs). Additionally, the researchers linked 5FA with L peptide (RLLDTNRPLLPY), a ligand for CS-GRP78, which enhances the uptake of rapamycin through the mTORC1 pathway. These “Hydra-ELPs” have been found to increase the potency of rapamycin and enhance its ability to sensitize cancer cells when targeting the cell surface form of GRP78 [78]. Polymeric nanoparticles carrying a reactive peptide for HSPA5 and loaded with Tx have been shown to slow down the growth of solid tumor cells in vivo and enhance the apoptosis of these cells when exposed to radiation [79]. Mab GRP78-Nanoparticles (NPs) have been shown to improve the effectiveness of the chemotherapy drug 5-fluorouracil in CS-GRP78 overexpressed human hepatocellular carcinoma cells. The NPs can effectively enter these cells and inhibit their growth and survival, leading to cell death by activating caspase 3. Concisely, by targeted drug delivery, Mab GRP78-NPs inhibit cancer cell invasion and ameliorate antitumor efficiency [80]. As presented here and in Table 1, upregulation of CS-GRP78 is generally associated with chemoresistance. Therefore, directly targeting CS-GRP78 is often the most effective method for achieving an anti-cancer effect and overcoming chemotherapy resistance (Fig. 1).

**Table 1:** Chemoresistance model that modulates CS-GRP78 expression

Chemotherapy drugs	Resistance model	Reference
Gemcitabine Doxorubicin Paclitaxel Cisplatin Bortezomib Rapamycin 5-Flurouracil	Pancreatic cancer Breast cancer Prostate cancer Lung cancer Prostate Cancer Prostate Cancer Hepatocellular Carcinoma	[42] [43] [41] [20] [77] [113] [80]

### Role of CS-GRP78 in tamoxifen resistance

The resistance to the hormonal therapy drug Tamoxifen presents a significant obstacle in treating estrogen receptor-positive breast cancer, potentially resulting in disease recurrence and poor outcomes. Hence, pinpointing molecular pathways that can target tamoxifen-resistant breast cancer cells is imperative. An increase in CS-GRP78 levels marks the development of tamoxifen resistance in breast cancer cells. The clinically relevant MCF7-LR breast cancer cells, which are tamoxifen-resistant, offer a model for examining the interaction of CS-GRP78 with its associated proteins.

In MCF7-LR cells, CD44v directly binds to CS-GRP78 in the plasma membrane nanodomains. Using a CS-GRP78 antibody reduces CD44v cell surface expression and suppresses cell spreading, revealing a new mechanism by which CS-GRP78 regulates tamoxifen-resistant breast cancer cells [81]. The COOH-terminal polyproline sequence of GRP78 was found to modulate STAT3 activation and enforced expression of the COOH-terminal peptide of GRP78 (amino acids 631–650) in MCF7L-R cells, leading to decreased CD44v levels, increased apoptotic markers, and reduced cell viability. This implies that enforcing the expression of the GRP78 COOH-terminal peptide may interfere with its interaction with CD44 and other proteins driving tamoxifen resistance, offering a new strategy for overcoming endocrine resistance in breast cancer. The expression of a short peptide with the COOH-terminal PRR region of GRP78 also reduces CD44v and Cyclin D1 protein levels and cell viability and increases apoptotic signaling. This suggests that the COOH-terminal PRR of GRP78 is crucial for its interaction with CD44v at the cell surface and enforcing its expression may offer a new strategy for reducing tamoxifen-resistant breast cancer cell survival. The COOH-terminal polyproline sequence of GRP78 is a unique characteristic that evolved in higher eukaryotes with uncharacterized signaling functions in controlling GRP78 cell surface expression and STAT3 activation in tamoxifen-resistant breast cancer cells [82].

VER-155,008 is an adenosine derived (ATP mimetic) small molecule with high affinity to the ATP site of GRP78. Through exposure to VER-155,008, the sensitivity of canine osteosarcoma cells to cytotoxic agents was decreased due to an increase in the levels of both HSP70 and GRP78, thus its functioning as a dual inhibitor [83]. When VER-155,008 inhibits GRP78 expression and in turn amplifies tamoxifen-induced apoptosis. On the other hand, if GRP78 is overexpressed, it makes the cells more resilient to tamoxifen-mediated cell death [84]. Inhibition of CS-GRP78 has been demonstrated to improve the response to tamoxifen resistance, which can lead to improved patient outcomes.

### Role of CS-GRP78 in radiotherapy resistance

CS-GRP78 expression in cancer cells is instrumental in radio-resistance, recurrence, and cell survival. Antibodies targeting CS-GRP78 can repress proliferation, induce cell death, and suppress PI3K/AKT/mTOR signaling in irradiated NSCLC and GBM cells. Further, enhancing the effectiveness of radiotherapy by combining it with anti-GRP78 antibodies may lead to better outcomes for patients with GBM or NSCLC [85]. Clarifying further, targeting CS-GRP78 with C38 Mab improves the effectiveness of radiation therapy by increasing radiosensitivity and reducing the motility and invasiveness of pancreatic ductal adenocarcinoma (PDAC) cells. Additionally, CS-GRP78 activates the transcriptional coactivators YAP and TAZ in a Rho-dependent manner, which amplifies the expression of target genes (Ctgf, Cyr61 and Axl) and promotes migration and radiation resistance in PDAC cells. Therefore, using C38 Mab in combination with radiation therapy may be a promising approach for treating PDAC [18]. Similarly, a triptolide bioactive component found in a Chinese medicinal herb called *Tripterygium wilfordii Hook F* is known to reduce the GRP78 expression level in leukemic and radio-resistant nasopharyngeal carcinoma cells [86].

Expressing GRP78 on the cells surface promotes radiation resistance and increases metastasis in HNSCC cells. Additionally, radiation causes an increase in the amount of GRP78 present in extracellular vesicles (EVs), which can transfer GRP78 to non-irradiated cells, potentially contributing to a bystander effect that leads to increased radiation resistance and metastasis in these cells. This suggests that EV-mediated transfer of GRP78 may play a significant role in the radioresistance and migration of HNSCC cells [13]. By targeting CS-GRP78, researchers could reduce the self-renewal and resistance to radiation treatment in a type of brain tumour stem cells called mesenchymal glioma stem cells (MES GSCs). This was accompanied by decreased activity of specific signalling pathways (STAT3, NF-κB, and C/EBPβ) involved in cancer growth. Additionally, targeting CS-GRP78 also regulated the activity of β-site APP-cleaving enzyme 2 (BACE2) via lysosomal degradation, this resulted in reduced tumour growth and resistance to radiation in the MES GSCs [87].

**Fig. 2:**
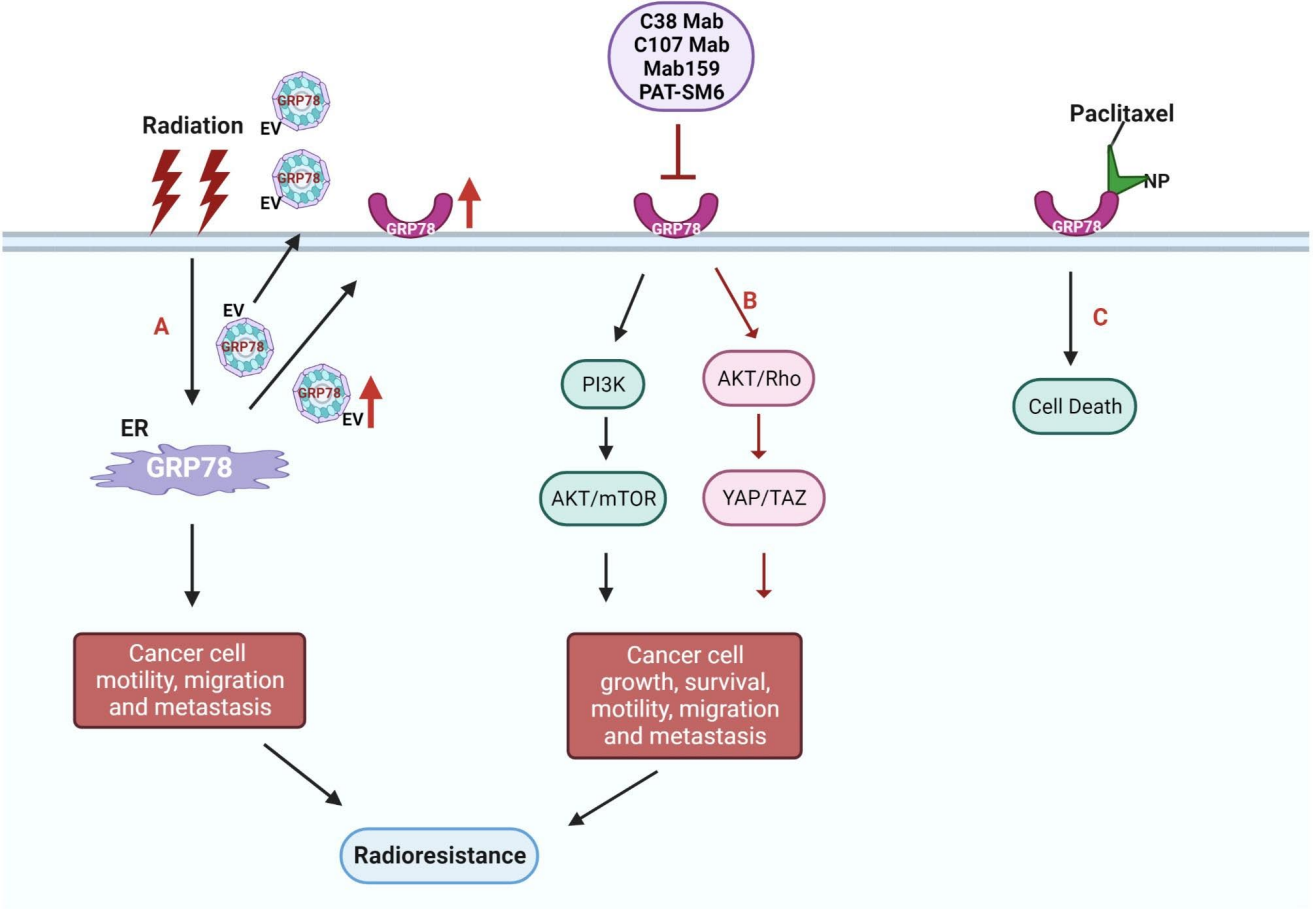
**Radioresistance mechanisms involving the CS-GRP78 axis and their therapeutic strategies.** (**A**) Radiation therapy further elevates cell surface expression of GRP78 level in resistant cancer cells and can also transfer GRP78 to non-irradiated cells through EVs to increase radio resistance through metastasis. Further investigation is required to understand the molecular mechanism by which radiation induces the translocation of GRP78 to the cell surface. (**B**) Illustrations summarizing the main interplay and radioresistance mechanism involving the CS-GRP78 signaling axis. (**C**) The CS-GRP78 targeting method utilizes a unique recombinant peptide and paclitaxel-containing nanoparticle that specifically target tumors that have been irradiated, resulting in higher levels of apoptosis and a greater delay in tumor growth compared to conventional chemotherapy approaches

When ionizing radiation (XRT) is applied to tumors and their blood vessels, it causes GRP78 to be transported to the surface of the cells. The nanoparticle-GIRLRG delivery system then explicitly delivers the chemotherapy drug Tx to the area affected by radiation. By utilizing the controlled, sustained drug release of the nanoparticle and the GIRLRG targeting peptide, the chemotherapy can be specifically directed to the XRT-induced CS-GRP78 receptor, resulting in significant cancer cell death. Remarkably, after a single administration of the nanoparticle- GIRLRG complex, Tx can be detected in irradiated tumors for up to 3 weeks, leading to an increase in apoptosis and slowing tumor growth. These results indicate that XRT treatment causes the expression of CS-GRP78, highlighting its role in the cellular stress response, and in cancer cell’s ability to evade the stressors that would lead to the death of normal cells. Thus, the targeting agent combines a new recombinant peptide with a nanoparticle that contains Tx, specifically targeting tumors that have been irradiated. This leads to a higher increase in apoptosis and a more significant delay in tumor growth compared to traditional chemotherapy methods [79]. Accordingly, inhibiting the radiation induced CS-GRP78 signaling axis in the recipient cells might promote anticancer effects and overcome resistance to radiotherapy. Anti-GRP78 antibodies that specifically target CS-GRP78 show promising co-targeting opportunities altogether (Fig. 2).

### Role of CS-GRP78 in drug resistance

Although expression of CS-GRP78 was consistently present, it increased with the progression of the disease and was significantly elevated in patients with drug and therapeutic resistance. Combining the broad-spectrum Bcl2 family inhibitor Obatoclax (OBX) and the Smac mimetic LCL161 leads to synergistic cytotoxicity and anti-proliferative effects on a wide range of human multiple myeloma (MM) cell lines. LCL161 blocks the function of IAPs, activating caspases, and inducing apoptosis, whereas OBX regulates the binding of pro-apoptotic and anti-apoptotic Bcl2 proteins and activates BH3-only proteins, leading to apoptosis. Additionally, both OBX and LCL161 impact the ERS response and activate specific branches of the UPR pathway. OBX activates the PERK/peIF2α/ATF4 and ATF6 branches and may contribute to pmTOR inhibition and protective autophagy. However, it also causes cell surface localization of GRP78, activates AKT through a PI3K dependent mechanism, and causes resistance to cell death. Conversely, LCL161 reduces Xbp1 splicing, hinders OBX-induced pAKT, and increases cells sensitivity to OBX, preventing OBX’s downregulation of pS6. As a result, the combination of LCL161 and OBX induces synergistic apoptosis in MM cells through multiple mechanisms [88]. Additionally, using inhibitors or knocking down IGF1-R suppresses GRP78 expression, which translocates from the ER to the cell surface. This facilitates physical interaction between GRP78 and IGF1-R [24]. Consequently, ONC212 (a fluorinated imipridone), a GRP78 modulator, and AG1024 (IGF-R inhibitor), induce a synergistic anti-cancer effect with apoptotic PANC-1 cells that survived each drug individually [89]. The interaction between GRP78 and IGF1-R molecules suggests that using GRP78 inhibitors to inhibit IGF-1R signaling in cancer cells may be viable[24].

Tumor cells can adapt to stressful environments through intercellular communication, the Transmissible ERS (TERS). BTZ and Tx enhance TERS signaling, which precipitated a UPR in human prostate cancer cells, including the surface expression of the chaperone GRP78. TERS-induced CS-GRP78 regulates cytoprotection and enables cells to adapt to nutrient and chemotherapy stress. As a result, TERS-mediated CS-GRP78 signaling in cancer cells promotes survival and drug resistance in cancer cells [41]. Drug-induced cell surface translocation of GRP78 is a promising and encouraging approach to promote anticancer effects, though uncommon, these are potent examples. Recent studies have revealed that GRP78 plays a role in mediating resistance to proteasome inhibitors (PI) in MM by promoting the formation of autophagosomes, a compensatory process for restoring protein degradation when the proteasome is blocked [90, 91]. This has been previously reported for the BRAF600E inhibitor vemurafenib in melanoma [92]. However, MM has shown increased sensitivity to PI when GRP78 is eliminated through drugs or shRNA [62, 71, 72].

Additionally, MM cells resistant to PI treatment have been found to have increased expression of GRP78 protein. In MM patients, GRP78 expression is associated with progressive disease [91]. These results align with previous studies on solid tumors, which have demonstrated a correlation between GRP78 expression, disease stage, invasiveness, and drug resistance [93]. Furthermore, cells from solid cancers often move GRP78 from the cytosol to the cell surface under stress for reasons that are not yet understood [7]. Therefore, GRP78 expressed on the cell surface of MM, can serve as a target for immunotherapy [94] with more efficacy.

The effect of CS-GRP78 on drug resistance was studied in a case report of a patient treated with PAT-SM6. The patient had a deteriorating condition while receiving treatment with a combination of BTZ, lenalidomide (len), DOX and dexamethasone, demonstrating resistance to all these drugs. Treatment with a reduced regimen of len and BTZ along with PAT-SM6 resulted in rapid response in both intra- and extramedullary lesions. However, the response was short-lived, highlighting the potential of PAT-SM6 as an antibody therapy targeting GRP78. However, it should be noted that this study only involved one patient and further controlled studies are needed to investigate response rates and optimal dosage regimens of this combination therapy. The high expression of CS-GRP78 caused by late-stage multidrug-resistant disease and concurrent len treatment led to significant cell death induced by PAT-SM6 via the induction of apoptosis. However, more research is needed to fully understand the role of soluble GRP78 in drug resistance and the impact of PAT-SM6 on the UPR [95]. Apart from, the mechanism of resistance to PAT-SM6 combination therapy, upregulation of CD55 and CD59 protective molecules could conserve cells from complement-mediated lysis as noticed for daratumumab [96]. In the future, studies are warranted to expound on the biological role of CS-GRP78 and find more strategies to prevail over drug resistance in MM.

What remains a challenge is the Multidrug resistance (MDR) to the treatment of gastric cancer (GC) GMBP1 (Gastric cancer MDR cell-specific binding peptide), ETAPLSTMLSPY that could adhere to the surface of GC MDR cells and reverse their MDR phenotypes. GRP78, an MDR-related protein, was recognized as a receptor of GMBP1. Targeting GMBP1 in MDR cells declined MDR1, Bcl-2 and GRP78 expression but increased the expression of Bax. However, suppressed GRP78 expression restrained MDR1 expression [97]. GMBP1 peptide also modulates the expression of EIF4E and MDR1 through the PI3K/AKT pathway [98]. Therefore, GMBP1 may make chemotherapy more effective against cancer cells that have developed resistance, known as GC MDR cells. This may be due to GMBP1’s ability to reduce the expression of GRP78 and MDR1. These findings suggest that GMBP1 may bind to a specific receptor for GRP78 and influence the MDR phenotype of the cells. All these ensure a new research perception on managing MDR in GC cells. The first peptide-drug conjugate PEP-DOX is competent in killing chemotherapy-resistant B-lineage ALL cells by targeting the surface expressed HSPA5 and constitutively active anti-apoptotic UPR pathway. Nonetheless, SYK has been identified as a new regulator of the anti-apoptotic UPR pathway, and it can selectively activate the expression of the HSPA5 gene. When HSPA5 over-expression negatively affects cancer treatment outcomes, targeting the link between the UPR and SYK kinase pathways with rationally designed SYK inhibitors may be effective. Studies suggest that the SYK P-site inhibitor C-61 is effective in treating leukemia partly due to its ability to turn off the anti-apoptotic aspect of the UPR pathway dependent on HSPA5 [99]. Additionally, chemotherapy-resistant B-lineage ALL cells are highly sensitive to a DOX-conjugated cyclic cell-penetrating peptide called PEP-DOX, which targets HSPA5 molecules on the cell surface [73]. Hormonal therapy-resistant breast and prostate cancer cells enhance the surface expression of GRP78, which can be exacerbated by conditions that trigger ERS. The GRP78 binding peptide is a remarkably enterprising molecule that can be used to deliver an anticancer agent. A 7-residue peptide L-VAP (SNTRVAP) has a strong ability to bind to the GRP78 protein, which is often overexpressed on glioma, glioma stem cells, vasculogenic mimicry and neo vasculature. By attaching L-VAP to a drug delivery system called micelles, it is possible to specifically target the drug to these cancer cells, potentially improving the effectiveness of the drug Tx in treating glioma [100].

This article and Table 2 outline that, drugs can increase the endogenous GRP78 may boost cell survival. This would be undesirable for cancer cells as it could hinder their elimination. Evidence suggests that drug induced GRP78 in tumor cells translocates into the surface leading to a better survival rate and resistance against treatments. This could also be a potential indicator for the metastasis of malignant cells. Therefore, CS-GRP78 expression in patients with tumors harboring drug resistance may be susceptible to clinical combinations that include CS-GRP78 Mab.

**Table 2:** Anti-cancer drugs mediate resistance through CS-GRP78.

Drugs	Target	Reference
**Obatoclax (OBX)**- BCL2 family inhibitor	Increases CS-GRP78 and their downstream signaling	[88]
**LCL161-** Smac mimetic	Blocks OBX induced AKT activation through CS-GRP78	[88]
**ONC212**-A fluorinated imipridone	Increases CS-GRP78 expression and IGF-R expression	[24]
**AG1024**-IGF-R inhibitor	Synergy with ONC212	[24]
**Vemurafenib**-BRAF inhibitor	Regulates subcellular localization of BRAF and GRP78	[92]
**C-61** -SYK P-Site inhibitor	Turns off anti-apoptotic aspects of GRP78	[99]

## Discussion

GRP78 on the cell surface may be a viable target for precision drug delivery. Precision therapy aims to deliver a high amount of medication to a specific organ or cell type while minimizing the risk of side effects by targeting a unique molecular entity. This can be achieved by attaching a drug or drug-loaded carrier to a ligand that specifically binds to a molecular target uniquely or highly expressed on the desired cells. CS-GRP78 based drug delivery system for cancer therapy is shown in Table 3.

**Table 3:** Various drug delivery carriers are used for CS-GRP78.

CS-GRP78 targeted delivery methods	Reference
**SP94-DOX-NDs (**SP94 peptide modified Doxorubicin loaded ultrasonic Nanodroplets)	[55]
**NPs-Tx-KDEL** (anti-KDEL functionalized polymerized nanoparticles loaded with Paclitaxel)	[67]
**RI-VAP-NLC/BTZ** (Nanostructured Lipid Carriers retro in verso isomer of I-VAP (SNTRVAP) RI-VAP with bortezomib)	[76]
**RI-VAP-micelles/paclitaxel (**Paclitaxel loaded polymeric micelles)	[100]
**L-5FA-RAPAMYCIN** (5FA with rapamycin binding domain linked with elastin like polypeptides (ELPs) L-peptide (RLLDTNRPLLPY))	[78]
**mab-GRP78-NPs** (Nanoparticles conjugated with antibody against GRP78)	[80]
**Paclitaxel NP with GIRLRG** (Recombinant peptide with paclitaxel encapsulating nanoparticle)	[79]
**Pep-DOX** (Doxorubicin conjugated Pep42)	[73]

Polymeric nanoparticles (NPs) are drug carriers that encase the drug in a polymeric matrix. NPs have a high loading capacity, allowing for the formulation of poorly soluble drugs as injectable suspensions. The surface of the nanoparticles can be modified with specific ligands such as antibodies, aptamers, glycoproteins, lectins, or peptides to target a specific cell or organ. Tx is a chemotherapy drug from the taxane family, commonly used to treat advanced and recurrent prostate cancer. Its low water solubility requires using cosolvents such as Cremophor®, which can cause severe side effects. NP formulation has been used to overcome the solubility problem associated with Tx. Recently, Tx-loaded NPs have been functionalized with Herceptin®, a targeting agent, for delivery to ovarian cancer cells that overexpress HER2 specific antigens. The efficacy of this drug delivery system has been reported in vitro and in vivo using the SKOV-3 cell model [101, 102]. Therefore, GRP78, expression on the surface of cancer cells, can be conjugated into chemotherapeutic drugs and nanocarriers, helping these agents to reach the target tissue and cells more easily. Recent studies have demonstrated that GRP78 is a potential target for Chimeric Antigen Receptor T (CAR T) cell therapy in acute myeloid leukemia (AML). The outcome of both studies indicated that GRP78-CAR T cells could effectively eliminate malignant cells in vitro and in vivo utilizing the same Pep42 peptide to construct the CAR T [103, 104]. Additionally, these cells were able to significantly eliminate primary AML blasts, indicating that a significant portion of AML patients may benefit from GRP78-CAR T cell therapy in the future. Importantly, these cells did not show cytotoxicity towards normal bone marrow cells or hematopoietic stem cells (HSCs). Furthermore, dasatinib during post-activation/transduction in T cells reduces CS-GRP78 expression, likely through inhibition of Src family kinases, further enhancing the efficacy of GRP78-CAR T cells [104]. Together, these studies confirmed that GRP78-CAR T cells are an effective and safe option for AML treatment, making GRP78 an attractive target for CAR T cell therapy. Thus, there are promising advances in using CS-GRP78 as a target for drug and gene delivery as well as immunotherapy to enhance efficacy and reduce the adverse effects of current cancer therapies.

Antibody-based therapies show promise for drug development in the central nervous system (CNS), but the blood-brain barrier (BBB) can prevent the entry of large molecules. GRP78-IgG, a type of antibody found in patients with cancer and autoimmune diseases, can serve as a valuable biomarker for early cancer detection and may also open the BBB for the delivery of treatments for CNS diseases [105–107]. Recent research has shown that GRP78-IgG can promote BBB transit of large-molecule therapies for CNS diseases and cancer. These suggest that CS-GRP78 plays a crucial role in BBB permeability [108] and is overexpressed in cancer cells, making it an attractive target for chemotherapy [18, 109, 110]. The combination of C38 Mab and radiotherapy could target cancer cells specifically, thereby preventing harm to normal tissue and reducing the likelihood of radioresistance and locoregional cancer recurrence [93, 111]. Drug resistance in solid tumors is a significant issue, and ERS is a contributing factor. The UPR, which regulates pro-survival or pro-apoptotic signals through three sensors, is significantly involved in drug resistance, although the mechanisms are not yet fully understood. This review highlights a possible connection between drug-induced ERS and the CS-GRP78 signaling axis.

Currently, we are investigating the mechanism of CS-GRP78 in cells resistant to bromodomain inhibitors to understand how to overcome drug resistance. Additionally, targeting CS-GRP78 and combining it with anticancer drugs could enhance drug sensitivity. Moreover, CS-GRP78 is a host factor that plays an essential role in allowing pathogens, such as viruses, in entering and infect cells, so inhibition of GRP78 may also provide potential therapeutic benefits when treating certain bacterial and viral infections [17, 112]. Molecularly targeted therapies for cancer driver pathways have demonstrated that tumors can develop inherent and acquired resistance, in preclinical and clinical studies. Solid tumors comprise a diverse mix of cancer, immune, and stromal cells, thus combination therapies are often necessary to achieve lasting responses.

CS-GRP78 is a crucial regulator of cellular responses to external stimuli and a buffer against various forms of cellular stress, including therapeutic interventions. It is a promising target for various oncogenic processes and resistance mechanisms. Targeting CS-GRP78 with C38 Mab is likely more effective when used as combination therapy, mainly if tumor cells rely on stress triggered by anticancer treatment. In addition, CS-GRP78 downstream target genes like c-MYC and anti-GRP78 Aab in patients’ serum can be used as robust biomarkers to identify patients with tumors. The survival and growth of these tumors are driven by CS-GRP78 dependent signaling axis either through intrinsic or extrinsic mechanisms, which might steer the finest use of C38 Mab in the clinic.

## Conclusion and future perspective

Systemically administered drugs can have unwanted effects on tissues other than the target, limiting their efficacy and increasing toxicity. Site-specific drug delivery could reduce off-target effects, decrease unwanted toxicities, and enhance a drug’s therapeutic efficacy. Intriguingly, CS-GRP78 is a promising biomarker for cancer because it is highly expressed on the surface of cancer cells but not on normal cells. This makes them a good target for imaging and therapy. Further, CS-GRP78 can carry drugs across the BBB to the tumor and into the brain. Consequently, CS-GRP78 could be an emerging concept for tissue-specific drug delivery approaches and their clinical translation.

Additionally, CS-GRP78 is associated with poor prognosis in several types of cancer, suggesting that it may help to predict patient outcomes. Unfortunately, research on CS-GRP78 in human studies has been relatively limited compared to the tools available in preclinical models. Therefore, parallel CS-GRP78 investigations between rodents and humans are highly warranted. This approach may help to close the translational gap between clinical and preclinical studies and provide CS-GRP78 as a biomarker that will significantly improve early detection, prognosis, and prediction of treatment response.

## Data Availability

Not applicable.
